# Biological Responses of the Coral *Montastraea annularis* to the Removal of Filamentous Turf Algae

**DOI:** 10.1371/journal.pone.0054810

**Published:** 2013-01-23

**Authors:** Neidy P. Cetz-Navarro, Julio Espinoza-Avalos, Héctor A. Hernández-Arana, Juan P. Carricart-Ganivet

**Affiliations:** 1 El Colegio de la Frontera Sur, Chetumal, Quintana Roo, México; 2 Unidad Académica de Sistemas Arrecifales, Instituto de Ciencias del Mar y Limnología, Universidad Nacional Autónoma de México, Puerto Morelos, Quintana Roo, México; Leibniz Center for Tropical Marine Ecology, Germany

## Abstract

Coral reef degradation increases coral interactions with filamentous turf algae (FTA) and macroalgae, which may result in chronic stress for the corals. We evaluated the effects of short (2.5 month) and long (10 month) periods of FTA removal on tissue thickness (TT), zooxanthellae density (ZD), mitotic index (MI), and concentration of chlorophyll *a* (Chl *a*) in *Montastraea annularis* at the beginning and end of gametogenesis. Ramets (individual lobes within a colony) consistently surrounded by FTA and ramets surrounded by crustose coralline algae (CCA) were used as controls. FTA removal reduced coral stress, indicated by increased TT and ZD and lower MI. The measured effects were similar in magnitude for the short and long periods of algal removal. Ramets were more stressed at the end of gametogenesis compared with the beginning, with lower ZD and Chl *a* cm^−2^, and higher MI. However, it was not possible to distinguish the stress caused by the presence of FTA from that caused by seasonal changes in seawater temperature. Ramets surrounded by CCA showed less stress in comparison with ramets surrounded by FTA: with higher TT, Chl *a* cm^−2^ and ZD, and lower MI values. Coral responses indicated that ramets with FTA suffered the most deleterious effects and contrasted with those measured in ramets surrounded by CCA. According to published studies and our observations, there could be at least six mechanisms associated to FTA in the stress caused to *M. annularis* by FTA. Owing to the high cover of FTA (in contrast to macroalgae and CCA) in the Caribbean, the chronic stress, the overgrowth and mortality that this functional algal group can cause on *M. annularis* species complex, a further decline of this important reef-building coral in the Caribbean is expected.

## Introduction

Coral reef degradation can involve a dominance shift from live coral colonies to dead corals overgrown by algae [1], [2]. Coral reefs in the Caribbean basin are among the most severely degraded in the world, with a remarkable decrease in coral cover [3]–[5]. An increase in algal cover on degraded coral reefs has led to a high frequency and longer duration of interactions between corals and algae [2], [6], [7]. Besides, positive and negative interactions of algal functional groups, such as macroalgae, filamentous turf algae (FTA) and crustose coralline algae (CCA), with corals have been reported [8]–[10]. However, macroalgae and FTA are generally assumed to have negative effects [11]–[14], while CCA is thought to have minimal or no detrimental effects on corals [12]–[15].

FTA, which may be mixed with macroalgae, can directly or indirectly provoke coral stress. Symptoms of coral stress caused by the presence of those algae include hypoxia, tissue disruption, altered pigmentation [13], [16], [17], bleaching [13], increment in thickness of the diffusive boundary layer [18], a major shift in the bacterial communities in the algae-coral tissue interaction (including pathogenic microbes) [17], reductions in fecundity, photosynthetic performance and growth rate [13], [19]–[23], and mortality [16], [24]. Similarly, reductions in tissue thickness, zooxanthellae density and chlorophyll *a* concentrations were identified as symptoms of coral stress for the coral *Montastraea faveolata* in competition for space with FTA [24]. Additionally, the mitotic index of zooxanthellae increases when corals face different stressful conditions [25], [26]. In contrast, ramets of *Montastraea annularis* bordered by CCA showed no deleterious responses. Instead, they had a slightly higher photochemical efficiency, higher concentrations of dissolved oxygen (hyperoxia), and an increase in the diversity of coral-associated bacterial communities, including non-pathogenic microbes [17], [23].

In addition to the study of effects on corals due to the presence of algae, another approach has been to investigate the responses of corals to the removal of algae. Studies involving the removal of algae have separately evaluated coral responses over relatively short (e.g., 10–12 day to 2–3 month) [17], [21], [27] and long (e.g., several months to years) [28]–[30] periods. However, a comparison of responses to the removal of algae at different time scales has not been carried out for a coral species.

*Montastraea annularis*, one of the most important reef-building coral species in the Caribbean [31], [32], is a hermaphrodite species with an annual reproductive cycle that begins in May and ends in August-September. The formation of gametes is asynchronous (i.e., formation of the female gametes begins in May and the formation of male gametes begins in June), while their release occurs synchronously in summer [33]. The formation of gametes is a fundamental process in the life history of corals, and there is evidence of a trade-off between reproduction and maintenance [34]. Thus, corals can divert their energy into reproduction instead of repairing injuries [35] or in other cases energy is shifted from reproduction to wound healing, with maintenance apparently limiting reproduction [36]. In addition, corals exhibit different responses depending on the state of the gametogenesis. For example, during fragmentation, corals with female gametes forming in the late reproductive state continued developing, while those in the early vitellogenic stage were resorbed [37]. A question arises whether at the end of the gametogenesis *M. annularis* diverts more energy to reproduction instead of competition for space with FTA, resulting in a more stressed coral.

In the Caribbean, the tissue of *M. annularis* is frequently surrounded and overgrown by FTA [38]–[40], a functional algal group of dense, multi-species assemblages of filamentous algae and cyanobacteria that grows faster and occupies available space faster than macroalgae [23], [24]. FTA, in combination with sediments, are considered a source of stress for corals when both are in contact [24], [41].

We evaluated the effects of FTA removal on *M. annularis* stress by measuring tissue thickness, zooxanthellae density, mitotic index and chlorophyll *a* concentrations over relatively short (2.5 months) and long (7 and 10 months) periods of time, and at the beginning and end of coral gametogenesis. Unmanipulated ramets that were permanently surrounded by mixed FTA or CCA (Rhodophyta, Corallinaceae) were used as experimental controls. Our working hypotheses were that, in comparison to the control ramets surrounded by FTA:

- The removal of FTA would reduce coral stress.
- The stress reduction would be larger with long versus short periods of FTA removal; and
- Ramets with FTA removal and the controls with CCA in their periphery would have lower stress.
- Moreover, coral tissue stress would be greater at the end than the beginning of the gametogenesis.

## Materials and Methods

### Study Site

**Figure 1:**
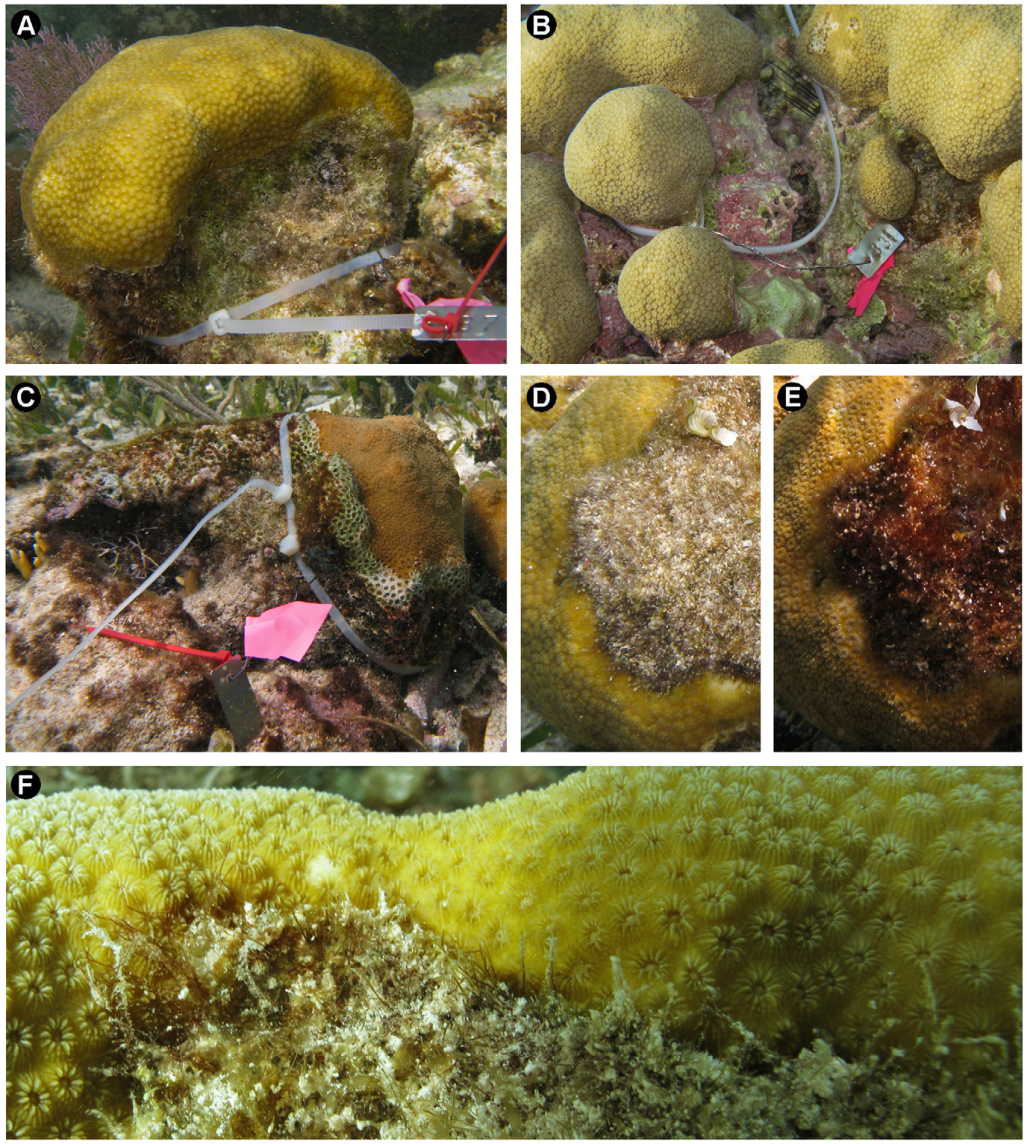
*Montastraea annularis* with and without surrounding algae, and algal mat without sediment. A) *M. annularis* ramet bordered by filamentous turf algae, B) *M. annularis* ramets bordered by crustose coralline algae, C) *M. annularis* ramet following the removal of filamentous turf algae, D) *M. annularis* ramet surrounded by filamentous turf algae with sediment trapped in the algal mat, E) ramet from Figure 1D following the removal of sediment from the algal mat using pressured air, and F) magnified view of the periphery of *M. annularis* being overgrown by filamentous turf algae through projections of prostrated axes. Photo credits: A–E by H Bahena-Basave and F by J Espinoza-Avalos.

The experimental study was performed at Xahuayxol (18° 30’ 11.9” N, 87° 45’ 24.8” W), located in the southern part of Quintana Roo in the Mexican Caribbean. The study site is close to the breaker zone in a reef lagoon at a depth of about 1.5 m. Two distinct forms of coral-algae interaction were observed at the study site: ramets (single lobes of a colony or genet, see [42]) of *M. annularis* surrounded by FTA at the periphery of their base (Figure 1A) and ramets surrounded by CCA (Figure 1B). The second type of interaction was likely facilitated by a high abundance of the black sea urchin *Diadema antillarum,* which has also been reported in other Mexican Caribbean reefs [43]. Mats of FTA (∼8 mm in height), with more than 50 intermixed species, predominantly consisted of creeping Rhodophyta but also included Cyanobacteria, Chlorophyta and Phaeophyceae species. Abundant sediment grains less than 0.3 mm in diameter were trapped within the mats. Conspicuous species in the mats of FTA were *Polysiphonia scopulorum* v. *villum*, *Lophosiphonia cristata*, *Herposiphonia bipinnata* (Rhodomelaceae), *Parviphycus trinitatensis* (Gelidiellaceae), *Centroceras clavulatum*, *Ceramium* spp. (Ceramiaceae), *Anotrichium tenue* (Wrangeliaceae), *Padina* sp. (*Dictyerpa* stage; Dictyotaceae), *Bryobesia johannae* (Cladophoraceae), *Lyngbya* spp. (Oscillatoriaceae) and *Dichothrix* spp. (Rivulariaceae).

### Experimental Design

**Figure 2:**
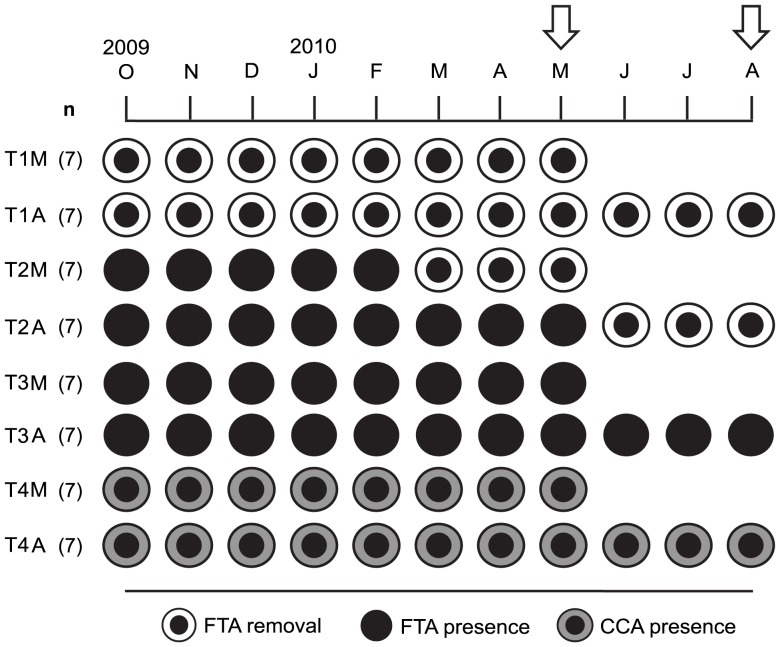
Graphic representation of the ramets used in the four treatments. Treatments included *Montastraea annularis* ramets with monthly/fortnightly removal of filamentous turf algae (FTA) surrounding the coral tissue (FTA removal), ramets with FTA in contact with the coral tissue (FTA presence), and ramets with crustose coralline algae (CCA) in contact with the coral tissue (CCA presence). Ramets were collected in May 2010 and August 2010 (arrows) at the beginning and the end of the *M. annularis* gametogenesis cycle. Treatments: T1M and T1A = long-term treatment of FTA removal over 7 and 10 months, respectively; T2M and T2A = short-term treatment of FTA removal over 2.5 months in May 2010 and August 2010, respectively; T3M and T3A = control ramets in constant contact with FTA; and T4M and T4A = control ramets in constant contact with CCA. n = 7 collected ramets per date per treatment.

Removal of FTA from experimental ramets of *M. annularis* was performed for 2.5 (with fortnightly algal removal) and 7–10 months (with monthly algal removal from October to December 2009 and fortnightly removal from January to August 2010) to evaluate the effects of short- and long-term algae removal on the coral at the beginning (May 25, 2010) and end (August 24, 2010) of gametogenesis (Figure 2).

A 2–3 cm wide belt of FTA around the periphery of the coral tissue was manually removed (Figure 1C) using a small wire brush (about 1 cm wide) and a knife, beginning on October 26, 2009 (month 0) and ending on August 24, 2010 (month 10; Figure 2). The first experimental treatment (T1) represented long-term algal removal, which ended on May 25, 2010 (T1M, 7 months of removal; n = 7) and August 24, 2010 (T1A, 10 months of removal; n = 7) to evaluate the coral responses to algal elimination at the beginning and the end of the gametogenesis cycle of *M. annularis*, respectively (Figure 2). The short-term algal removal (2.5 months, T2) evaluated coral conditions at the beginning and end of the gametogenesis cycle and was performed from March 2010 to May 2010 (T2M; n = 7) and June 2010 to August 2010 (T2A; n = 7), respectively (Figure 2). The last two treatments were controls: non-manipulated ramets surrounded by FTA (T3) and CCA (T4) collected in May (T3M (n = 7) and T4M (n = 7)) and August 2010 (T3A (n = 7) and T4A (n = 7)) (Figure 2). In total, we collected 56 ramets (n = 7 ramets per treatment per date) from several colonies. As part of the experimental design, 112 ramets were tagged at the beginning of the study as a precaution in case of loss or injury of ramets due to experimental manipulation or natural disturbances. For each treatment, the ramets were identified using four different tags that were easily recognizable in the field with plastic cable ties fastened at the base of the ramets and stainless steel wire hitched to the tags (Figures 1A–1C). None of the tags or wires was in contact with the coral tissue. Control ramets were tagged in order to verify that the coral contact with FTA and CCA persisted throughout the experiments.

Because sedimentation alone is a stress factor for corals [13], [44], [45] and corals stressed by sedimentation may have a lower capacity to tolerate other stressors [46], such as FTA [13], we attempted to separate the stress due to FTA and sediments on *M. annularis* (Figure 1D). We included a treatment group that consisted of ramets subjected to the removal of sediment from the FTA (Figure 1E) by using pressure air with a blowgun attached to a dive tank. This treatment was not feasible because of the high rate of sedimentation that occurred at the study site. After removal, the sediments replenished within the FTA mat in 1–3 days. As a result, the required frequency of sediment removal was beyond our capabilities and was not pursued.

To evaluate the effects of short- and long-term algal removal on *M. annularis* at the beginning and the end of gametogenesis, we measured the tissue thickness (TT), zooxanthellae density (ZD), mitotic index (MI), chlorophyll *a* (Chl *a*) zooxanthellae^−1^ (Chl *a* zoox^−1^) and Chl *a* cm^−2^. Each experimental ramet was chiseled underwater and fragmented into one-half and two-quarter sections on the collection dates. The half ramet was used to evaluate the TT from the center. One-quarter of the ramet was used to obtain the blastate and to determine the ZD, MI, Chl *a* zoox^−1^ and Chl *a* cm^−2^, and the other quarter was used for fecundity evaluations that will be reported elsewhere. The collected fragments were deposited in labeled dark plastic bags filled with seawater and placed in coolers for protection from the sun and temperature changes during transport to the laboratory. The biological parameters from the *M. annularis* fragments were measured using the methods described by Quan-Young and Espinoza-Avalos [24]. The permit to collect the samples was provided by the Secretaría de Agricultura, Ganadería, Desarrollo Rural, Pesca y Alimentación (SAGARPA; permit number DGOPA.10745.121009.3629).

### Data Analyses

The biological parameter data from *M. annularis* were subjected to Kolmogorv-Smirnov and Levenés tests for normality and homogeneity of group variances, respectively. Two-way analysis of variance (ANOVA; factors: treatment and extraction date) and a post hoc test (Tukey's honest significant difference) were performed on untransformed data, with the exception of ZD in which the data were log transformed. Additionally, *a posteriori* power analyses (Several Means, ANOVA, 2-Way) were carried out for all data to compare mean coral responses for both factors. Because the TT samples from T1A were lost, only the data from three treatments (T2–T4) were used to analyze this biological parameter.

## Results

### Beginning Versus End of Gametogenesis in *Montastraea annularis* (Factor: Date)

**Figure 3:**
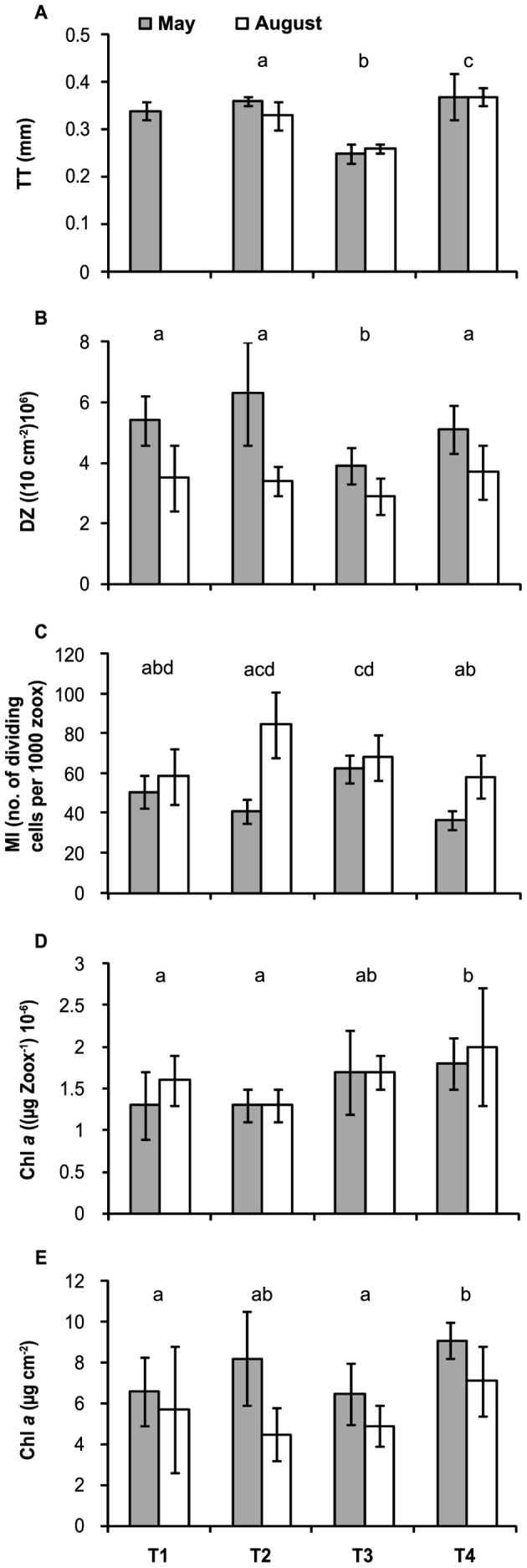
Biological parameters of *Montastraea annularis* per date per treatment. Mean values ± S.D. of A) tissue thickness (TT), B) zooxanthellae density (ZD), C) mitotic index (MI), D) chlorophyll *a* (Chl *a*) zooxanthellae^−1^ (Chl *a* zoox^−1^) and E) Chl *a* cm^−2^, per date (May and August) per treatment (T1–T4). Treatments: T1 = ramets with long-term removal of filamentous turf algae (FTA), T2 = ramets with short-term removal of FTA, T3 = control ramets with FTA in permanent contact with the coral tissue, and T4 = control ramets with crustose coralline algae in permanent contact with the coral tissue. Lower-case letters (a–d) above each pair of bars indicate treatments (combining both date values) that were significantly different from one another (α = 0.05). T1 of TT was not included in the analysis.

The mean values of TT and Chl *a* zoox^−1^ in the tissue of *M. annularis* were similar at the beginning (May) and the end (August) of the gametogenesis cycle (Table 1). Therefore, the null hypothesis of no time differences for both variables cannot be rejected, but the estimated power of the test was low due to variation in the pattern of responses within treatments. However, ZD and Chl *a* cm^−2^ were significantly higher in May than in August, while the MI was lower in May than in August (Table 1). The estimated power of the test was high, providing certainty of a real effect of the factor ‘date’, as can be observed from a consistent pattern of differences within treatments (Figure 3).

**Table 1:** Two-way analyses of variance evaluating the effects of sampling date and experimental treatment on biological parameters of *Montastraea annularis* ramets.

Source	df	MS	F	p	Conclusion (Tukey’s test)
**Tissue thickness**					
Date	1	0.000	1.31	0.26	ns
Treatment	2	0.051	165.37	0.00	T3 < T2 < T4
Date X Treatment	2	0.002	5.73	0.01	Significant (T3M < T2M, T4M; T3A < T2A < T4A and T2M > T2A)
**Zooxanthellae density**					
Date	1	0.466	62.16	0.00	May > August
Treatment	3	0.052	6.93	0.00	T3 < T1, T2, T4
Date X Treatment	3	0.013	1.67	0.19	ns
**Mitotic index**					
Date	1	5490.540	49.54	0.00	May < August
Treatment	3	932.754	8.42	0.00	T3 > T1, T4 and T4 < T2, T3
Date X Treatment	3	1060.445	9.57	0.00	Significant (T3M > T2M, T4M; T2A > T1A, T4A; T2M < T2A and T4M < T4A)
**Chl ** ***a*** ** Zoox^−1^**					
Date	1	2.23E-13	1.39	0.24	ns
Treatment	3	1.08E-12	6.74	0.00	T4 > T1, T2
Date X Treatment	3	9.61E-14	0.60	0.62	ns
**Chl ** ***a*** ** cm^−2^**					
Date	1	59.949	18.22	0.00	May > August
Treatment	3	15.217	4.62	0.01	T4 > T1, T3
Date X Treatment	3	5.440	1.65	0.19	ns

ns = not significant. Treatments are described in Figure 2.

### Presence or Absence of Algae in the Biological Parameters of *Montastraea annularis* (Factor: Treatment)

Ramets permanently surrounded by FTA (T3) showed the lowest mean values of TT and ZD, and high MI compared to T1 and T4 (Table 1, Figure 3). Interaction terms showed that lower values of TT were found at both sampling dates, as well as higher values of MI in T3M than in T2M (Table 1). When the ramets were consistently surrounded by CCA (T4), the Chl *a* (Chl *a* zoox**^−^** ^1^ and Chl *a* cm**^−^** ^2^) and TT were the highest and the MI was one of the lowest compared to the other treatments. The ramets surrounded by FTA and CCA had opposite responses in terms of TT, MI, ZD and Chl *a* cm**^−^** ^2^ (Figure 3, Table 1). The ramets surrounded by CCA and those with FTA removal for long (T1) and/or short (T2) periods had similar values of ZD and MI. However, different values between these treatments were found for TT (although interaction terms showed equal values in May) and both measurements of Chl *a* (Figure 3, Table 1).

### Long Versus Short Periods of Algal Removal on *Montastraea annularis*

The mean values of ZD, MI, Chl *a* zoox**^−^** ^1^ and Chl *a* cm**^−^** ^2^ from the tissues of the *M. annularis* ramets (Figure 3) were not significantly different (*P*>0.17, Tukey HSD; data not shown) between the long- and short-periods of algal removal. Thus, the effects were equivalent in the ramets following FTA removal for 7 to 10 months (T1) and 2.5 months (T2). This comparison was not possible for the TT in T1 due to loss of material.

## Discussion

As hypothesized, the removal of FTA from *M. annularis* reduced the stress on the coral, as indicated by increased TT and ZD in comparison to the control ramets surrounded by FTA. The increase in TT of ramets of *M. annularis* with FTA removal was similar in corals with the absence (in comparison to the application) of stressors like sedimentation, White syndrome, bleaching, turbidity and light attenuation [47]–[49]. The increase in ZD in ramets with FTA removal was also similar to measurements of this biological parameter in corals with the absence of stressors like low seawater temperature regimes, exposure to cyanide, sedimentation and Pink-line syndrome [26], [50]–[52]. Moreover, TT of corals has been suggested as a high-priority bioindicator for long- and short-term environmental chronic effects on corals [48], and the loss of zooxanthellae has been proposed as an indicator of stress in corals [53]. Lower values of MI were also found in ramets with FTA removal (T1), which is a condition similar to that shown in corals without application of stressors like exposure to cyanide, bleaching, elevated temperatures and Pink-line syndrome [50], [52], [54], [55].

Other member of the *M. annularis* species complex (*M. faveolata*) from our study area, with similar form and species composition of surrounding FTA, was stressed by competition for space with FTA [24], which reduced TT and ZD. In our study, the removal of FTA from *M. annularis* reduced the stress caused by the presence of FTA, increasing TT and ZD. The stress on *M. faveolata* caused by the presence of FTA also reduced the Chl *a* cm**^−^** ^2^ in its tissue [24]. However, the removal of FTA from *M. annularis* did not induce an increase in Chl *a* cm**^−^** ^2^, as expected, since this is a response of corals with absence of stressors like sedimentation, low seawater temperature regimes and bleaching [26], [51], [54]. The lack of consistency in Chl *a* cm**^−^** ^2^ results of both studies ([24] and this study) is attributed to the difference of methods used in both studies (manual FTA removal vs. reciprocal transplants of live and death colonies covered with FTA), particular conditions of study sites (Xahuayxol vs. Xcalak) and the *Montastraea* coral species under study (*M. annularis* vs. *M. faveolata*).

The positive responses of *M. annularis* to the removal of FTA, by increasing TT and ZD as well as reducing MI, are similar to other studies reporting beneficial coral responses to the removal of different functional groups of algae at different scales of space and time. Thus, the removal of macroalgae every 1.5–2.0 months for 2 years from patch reefs almost doubled the coverage, growth, and fecundity of *Acropora* spp. [28], while the removal (every 2–3 months for 6 months) of ∼2 cm of the brown algae *Lobophora variegata* from the basal tissue of *Porites cylindrica* noticeably diminished tissue mortality [30]. In addition, the removal (every 2 weeks for 2.5 months) of algal turfs and macroalgae surrounding *Porites astreoides* juveniles more than doubled the growth of the control colonies [27], and the clearance of macroalgae every 2 weeks for 3 months from the basal periphery increased the fecundity of *M. annularis* [21]. Finally, scrapping turf and fleshy macroalgae for 10–12 days from the edge of *M. annularis* tissue restored hyperoxia [17]. Nonetheless, two studies [28], [29] reported that some coral species did not display positive responses (in terms of coverage, growth, fecundity and mortality) to the removal of algae.

The reduction of stress on *M. annularis* with FTA removal during 2.5 or 7–10 months was similar, contrary to our hypothesis. We expected less stressed ramets under long- vs. short-periods of FTA removal. It is possible that the reduction of coral stress due to FTA removal, measurable in terms of the biological parameters that we evaluated, happens within 2.5 months of removal, and our later stress evaluation (i.e., 7–10 months) would be similar to the first (i.e., 2.5 months). For example, *M. annularis* restored hyperoxia in the tissue after 10–12 days of turf algae removal, but the recovery was not statistically significant for ramets bordered by algae [17]. Thus, the first detectable reduction of stress on *M. annularis*, for the biological parameters with which we evaluated stress, probably occurs between 0.5 and 2.5 months of FTA removal.

Ramets of *M. annularis* surrounded by CCA showed less stress in comparison with ramets surrounded by FTA, with higher TT, Chl *a* cm**^−^** ^2^ and ZD, and lower MI values. However, low stress of ramets surrounded by CCA and those with both periods of FTA removal was exhibited only in terms of ZD, while TT was similar to that in ramets with short-periods of algal removal, and MI similar to long periods of algal removal. Thus, those results support in part our hypothesis that ramets with CCA and FTA removal would have lower stress when compared with ramets surrounded by FTA. Certainly, our study confirms positive coral responses to the presence of CCA compared to stressful effects of FTA around the coral tissue. For example, CCA bordering coral tissue did not appear to be antagonistic and did not cause tissue disruption to the corals as opposed to FTA, which has been shown to increase exposure of corals to potential pathogens and virulent genes and to cause coral tissue disruption [13], [17]. Also, a lower rate of coral photosynthesis in CCA-coral than in FTA-coral interfaces has been predicted [18]. The reduction of coral stress observed in interactions with CCA (in comparison with other functional algal groups, such as FTA or macroalgae [21], [30], [56]) is an additional benefit of CCA on coral reefs. It is generally recognized that CCA contribute to coral recruitment, solidification of the reef framework and prevention of bioerosion of the coral substratum [15], [57], [58]. Even more, the abundance of coral-CCA interactions has been positively correlated with coral cover [17], and atolls in pristine condition are dominated by reef-building stony corals and CCA with abundant coral recruits [59].

Ramets of *M. annularis* surrounded by FTA were the most stressed, responding in an opposite manner to those bordered by CCA, with the lowest TT, ZD and Chl *a* cm**^−^** ^2^, and the highest MI. These results contrast with other studies [9], [60]–[62] that have shown minor or no effects of mixed turf algae on corals. However, our results are supported by similar findings observed in other reefs of the world [17], [18], including colonies of the *M. annularis* species complex [13], [24], [63], [64]. This study was not designed to identify the mechanisms by which FTA stress *M. annularis*, although we tried to separate the stress caused by the presence of FTA and sediments. However, from other studies it can be concluded that several mechanisms associated with FTA are involved in ramet stress of *M. annularis*: 1) FTA can directly overgrow coral tissue by extending their prostrated axes (Figure 1F; [63]), perhaps involving allelochemical effects [9]. 2) Algal filaments overgrowing corals can trap mucus from the coral and later, sediment (Figure 1F), apparently increasing the damage to the underlying tissues [9]. 3) Unattached FTA cushions projecting above the coral probably stress the coral tissue beneath the algae due to shading [24]. 4) FTA competing with corals may facilitate the invasion by virulent bacteria that compromise coral tissue [17]. 5) Cyanobacteria, such as *Dichothrix* spp. and *Lyngbya* spp., are found intermixed in the FTA [18], [65], [66] and may cause some degree of stress to the coral by allelopathy. *Lyngbya* spp. can cause severe damage to live coral tissue [20] and inhibit the recruitment of coral larvae [67]. 6) The sediments trapped in the species consortium of FTA attached to the periphery of the coral tissue may stress and kill coral through smothering and tissue burial [24], [41], [63], [64].

*Montastraea annularis* ramet stress increased at the end (August) in comparison to the beginning (May) of gametogenesis, with lower values of ZD and Chl *a* cm**^−^** ^2^, and higher values of MI, as expected. *M. faveolata* has a similar reproductive cycle as *M. annularis* [68] and, in a reef close to our study site, also showed lower values of Chl *a* cm**^−^** ^2^ and higher values MI in August than in May [24]. However, it has been speculated that lower values of ZD, Chl *a* cm**^−^** ^2^ and Chl *a* zoox**^−^** ^1^, occurring during summer-fall, are driven by seasonal changes in light and elevated temperature of seawater, and it was hypothesized that all tropical reef-building corals would exhibit that pattern [69]. Nonetheless, the hypothesized universal pattern is not shown within the species of the *M. annularis* complex and other species included in the study where the hypothesis emerged [69]. For example, and contrary to the assumed pattern, *M. faveolata* from our study area had high values of ZD and Chl *a* zoox**^−^** ^1^ in summer [24], while in a second study [70] both *M. annularis* and *M. faveolata* showed similar values of ZD in both summer and winter. Additionally, mean monthly sea surface temperatures (SST) over four years (2006–2009) from a nearby site (18° 30’ N, 87° 30’ W) ranged from 26.9°C to 29.6°C in May and from 28.5°C to 29.9°C in August (data provided by M. Eakin, NOAA). This relatively small range of SST between dates possibly tampered the stressing effects of temperature increments on the biological parameters of *M. annularis* we measured in August. Since the three *Montastraea* and the two *Acropora* species involved in [69] spawn in summer [32], [68], [71], the possible coral stress caused by reproduction and that caused by increasing SST cannot be distinguished. When stress caused by wounds and reproduction occur concurrently, corals divert more energy into reproduction [35], healing injuries, or both attributes at the same time with allocation of insufficient energy to either attribute [36]. Our experimental design cannot discriminate between environmental and reproductive factors (if they exist) to explain the differences in the biological parameters at the end of the reproductive cycle of *M. annularis*. However, it has been predicted that corals with metabolic imbalances under low stress levels and maximizing reproduction would tend to reduce the chlorophyll concentration and biomass of zooxanthellae [72], as we observed at the end of the *M. annularis* gametogenesis cycle.

In summary, our experimental results indicate that the removal of FTA reduced the stress in *M. annularis*. However, there was no difference in this reduction between long- and short-term FTA removal, probably because measurable stress caused by the presence of FTA in terms of the biological parameters we evaluated occurs before 2.5 months and responses beyond that period make no difference in terms of stress of *M. annularis* tissue. The reduction of coral stress was more evident when the ramets were surrounded by CCA, adding a positive coral response to the beneficial roles of this functional algal group to coral reefs. Our results could not distinguish differences in coral stress at the beginning and end of the coral reproductive cycle. Ramets surrounded by FTA were the most stressed ones, confirming the deleterious effects that this functional algal group exerts on corals from the Caribbean [17], [24] and other reefs from around the world [9], [73]. Certainly, in the Caribbean, the turf algal group is more abundant than other groups, such as macroalgae and CCA [39]. At first, turf algae stress members of the *M. annularis* species complex ([24] and this study), later overgrowing and killing its tissue [24]. The permanent presence of FTA constitutes a chronic source of stress for the *M. annularis* species complex, one of the most important reef-building species in the wider Caribbean [74], which provides high structural complexity to reef communities [33], [75], [76]. Serious declines of *M. annularis* cover (50–72%) have been reported from Caribbean sites [37], [77] in one decade, and further decline is expected if the predominant contact of their tissue with FTA and macroalgae do not change in the near future.
